# Immune mediators of sea-cucumber *Holothuria tubulosa* (*Echinodermata*) as source of novel antimicrobial and anti-staphylococcal biofilm agents

**DOI:** 10.1186/2191-0855-3-35

**Published:** 2013-06-24

**Authors:** Domenico Schillaci, Maria Grazia Cusimano, Vincenzo Cunsolo, Rosaria Saletti, Debora Russo, Mirella Vazzana, Maria Vitale, Vincenzo Arizza

**Affiliations:** 1 Department STEBICEF, Università degli Studi di Palermo, Via Archirafi, 32, Palermo, 90123, Italy; 2 Department Scienze chimiche, Università degli Studi di Catania, Viale A. Doria, 6, Catania, Italy; 3 Istituto Zooprofilattico Sperimentale della Sicilia “A. Mirri”, Via G. Marinuzzi, 3, Palermo, 90129, Italy; 4 Istituto Euro-Mediterraneo di Scienza e Tecnologia, Palermo, Italy

**Keywords:** Biofilm, Staphylococci, Antimicrobial peptides (AMP), Innate immunity

## Abstract

The present study aims to investigate coelomocytes, immune mediators cells in the echinoderm *Holothuria tubulosa*, as an unusual source of antimicrobial and antibiofilm agents. The activity of the 5kDa peptide fraction of the cytosol from *H. tubulosa* coelomocytes (5-HCC) was tested against a reference group of Gram-negative and Gram-positive human pathogens. Minimal inhibitory concentrations (MICs) ranging from 125 to 500 mg/ml were determined against tested strains. The observed biological activity of 5-HCC could be due to two novel peptides, identified by capillary RP-HPLC/nESI-MS/MS, which present the common chemical-physical characteristics of antimicrobial peptides. Such peptides were chemically synthesized and their antimicrobial activity was tested. The synthetic peptides showed broad-spectrum activity at 12.5 mg/ml against the majority of the tested Gram-positive and Gram-negative strains, and they were also able to inhibit biofilm formation in a significant percentage at a concentration of 3.1 mg/ml against staphylococcal and *Pseudomonas aeruginosa* strains.

The immune mediators in *H. tubulosa* are a source of novel antimicrobial peptides for the development of new agents against biofilm bacterial communities that are often intrinsically resistant to conventional antibiotics.

## Introduction

The survival and fitness of echinoderms in a marine environment, a habitat heavily populated by micro-organisms, suggest that they have developed a potent defensive mechanism that is part of an innate immune system (Tincu and Taylor 2004). The absence of fouling on many of these species indicates an effective defensive strategy to prevent the adhesion of bacteria, the first organisms that allow the formation of biofilm structures and fouling. With the aim of discovering new antimicrobial molecules, the echinoderm immune system was investigated as an unusual source of novel antimicrobial peptides (AMPs) with antibiofilm activity.

AMPs are small molecular weight proteins with broad spectrum antimicrobial activity against bacteria, viruses, and fungi. These evolutionarily conserved peptides are widely distributed in living organisms. Molecules with microbiocidal properties are found since in ameboid protozoa (Leippe 1999), prokaryotes (Pag and Sahl 2002), and plants (Garcia-Olmedo et al. 1998). Usually AMPs are positively charged and have both a hydrophobic and hydrophilic side that enables the molecule to be soluble in aqueous environments yet also enter lipid-rich membranes and kill target cells by using diverse mechanisms.

By focusing on the AMPs of echinoderms, many peptides have been discovered. In the coelomocytes (the immune mediators in echinoderms) of the sea star *Asterias rubens* a number of potential AMPs that are fragments of actin, histone HA2 and filamin were identified (Maltseva et al. 2004). In a recent study, the smallest peptide fragment of a beta-thymosin detected in the coelomocytes of Mediterranean sea-urchin *Paracentrotus lividus* showed the common chemical-physical characteristics of AMPs (Schillaci et al. 2012). In addition, two cystein-rich AMPs, named centrocyns, have been identified in the green sea urchin *Strongylocentrotus droebachiensis* (Li et al. 2010)*.*

Recently Haug et al. (2002) described an antimicrobial peptide in the supernatant of the egg homogenate of *Cucumaria frondosa* with a high antibacterial activity, especially against Gram-positive bacteria. In *Cucumaria echinata* a peptide of 20 amino acids was described, corresponding to the sequence from residue 332 of hemolytic lectin CEL-III, which exhibited strong antibacterial activity toward Gram-positive bacteria. The antibacterial activity of this peptide was correlated with its membrane-permeabilizing activity that perturbs bacterial cell membranes, leading to enhancement of their permeability (Hatakeyama et al. 2004).

Over the last years, the study on natural AMPs has attracted considerable interest as a new class of antimicrobial drugs with antibiofilm activity (Batoni et al. 2011; Herrmann et al. 2010).

The growth of bacteria as biofilms is responsible for biomaterial and device-associated infections (Donlan 2001). Furthermore it is an important virulence factor of pathogens, like *Staphylococcus aureus* and *Pseudomonas aeruginosa*, in the development of the chronic form of their infections in human beings, such as native valve endocarditis, burn or wound infections, cystic fibrosis-associated infections, otolaryngological infections, etc. (Post et al. 2007). Biofilms show multi-factorial resistance to conventional antibiotics. Currently no effective therapies that target microbial biofilms exist; the prompt removal of the contaminated device or surgical intervention remain the most effective means to treat biofilm-associated infections (Brady et al. 2008). Therefore novel anti-biofilm agents, treatments and strategies are needed (Projan and Youngman 2002).

With the aim of searching for new antimicrobial agents as alternatives to conventional antibiotics, we focused on the coelomocytes of the echinoderm *Holothuria tubulosa* (sea*-*cucumber). The antimicrobial activity of a 5kDa peptide fraction from coelomocyte cytosol of *H. tubulosa* (5-HCC) was evaluated against a group of important reference strains of well-known human pathogens. Nine peptides were detected in the content of 5-HCC, and two of them were chemically synthesized and tested for their antimicrobial and anti-biofilm activities.

## Materials and methods

### Animals and bleeding procedure

*H. tubulosa* (sea cucumber) were collected from the Gulf of Palermo (Mongerbino 38°06’58” nord, 13°30’26” est) at a depth of 10 m near a prairie of *Posidonia oceanica* and maintained until their use in a refrigerated aquarium at 15°C and fed with commercial invertebrate food (Algamac 2000 Bio-Marine CA USA). The coelomic fluid (CF) was collected by cutting the anterior-dorsal part of the animals with a scalpel and draining the fluid into a plastic cup containing an isosmotic anticoagulant solution (0.5 M NaCl, 20 mM Tris–HCl, 30 mM EDTA; pH 7.4) (ISO-EDTA). Coelomocytes were separated from plasma by centrifugation at 900 g for 10 min at 4°C, washed twice in ISO-EDTA and suspended in the same medium at 5 × 10^6^ cells/ml in ISO–EDTA. Total cell counts were determined using an improved Neubauer haemocytometer and dead cells were evaluated by using the eosin-y exclusion test (0.5% in ISO-EDTA).

### Preparation of coelomocyte lysate supernatant (CLS)

Acid-soluble protein extracts were prepared according to a previously described method (Mercado et al. 2005) with some slight modifications. Coelomocytes (1 × 10^7^) were suspended in a solution of 10% acetic acid in ISO without EDTA, sonicated (Sonifier Branson, model B-15 Danbury, CT USA) for 1 min at 0°C (1 pulse per second, 70% duty cycle) and centrifuged at 27,000 × g for 30 min at 4°C to remove any precipitate. After centrifugation, the supernatants were filtered (0.45 μM, Millex™, Millipore Corp.) and freeze dried to be later re-dissolved in H_2_O.

A 5 kDa peptide fraction of the cytosol from coelomocytes (5-HCC) was obtained by using a filter with a membrane with a nominal size of 5 kDa (Ultrafree-0.5 PBCC Centrifugal filter Unit, Amicon Millipore, MA, USA).

### Identification of 5kDa peptide fraction (5-HCC) components

#### ***Capillary RP-HPLC/nESI-MS/MS analysis***

HPLC-grade water and CH_3_CN were provided by Carlo Erba (Milan, Italy). Capillary RP-HPLC/nESI-MS/MS analysis of the in-gel enzymatic digests was performed using an Ultimate 3000 LC system combined with an autosampler and a 1:100 flow splitter (Dionex Corporation, Sunnyvale, CA, USA), coupled on-line with a linear ion trap nano-electrospray mass spectrometer (LTQ, Thermo Fischer Scientific, San Jose, CA). Ionization was performed with liquid junction using an uncoated capillary probe (20 mm i.d.; New Objective, Woburn, MA, USA). 2 mg of 5-HCC was freeze dried and re-dissolved in 0.1% aqueous formic acid (FA) (1 mg/ml). 10 μl of this solution was directly loaded onto a C18 μ-precolumn cartridge (0.3 μm × 5 mm, 100 Å, 5 μm, PepMap, Dionex) equilibrated with 0.1% aqueous formic acid (FA) at a flow rate of 20 μl/min for 4 min. Then, the solution was switched onto a reversed-phase C18 column (0.18 × 150 mm, 300 Å, 5 μm, BioBasic, ThermoFisher Scientific) and peptides were separated by elution at room temperature with a linear gradient of solvent B (ACN + 0.5% FA) in A (H_2_O + 0.5% FA) from 20% to 55% in 40 min at a flow rate of 1.5 μl/min. The nano-ESI source operated under the following conditions: capillary temperature 220°C, source voltage 2 kV, capillary voltage 45 V. Repetitive mass spectra were acquired in positive ion mode in the *m/z* range 450–2000. Characterization of peptide ions was performed by the data-dependent method as follows: 1) full MS scan (mass-to-charge ratio 450–2000); 2) zoom scan of the three most intense ions (isolation width: 2 Da) and 3) MS/MS of these three ions (activation Q 0.250; activation time 30 ms; normalized collision energy 27 a.u.). Mass calibration was made using a standard mixture of caffeine (Mr 194.1 Da), MRFA peptide (Mr 423.6 Da) and Ultramark (Mr 1621 Da). Data acquisition was performed using the Excalibur v. 1 · 4 software (ThermoFisher Scientific).

#### ***Database search and peptide identification via MASCOT Engine***

MS/MS data were used to perform peptide and protein identifications by searching in the NCBI non-redundant protein sequence database of *Echinodermata* (60697 entries, updated June 2011) using the MOWSE algorithm as implemented in the MS search engine MASCOT server (Matrix Science, London, UK, version 2.3). MASCOT was set up as follows: i) assuming no enzyme as cleavage specificity; ii) with a fragment ion mass tolerance of ±0.6 Da and a parent ion mass (PIM) tolerance of ± 1.6 Da; iii) specifying oxidation of methionine, transformation of N-terminal glutamine and N-terminal glutamic acid residue in the pyroglutamic acid form as variable modifications.

In MASCOT searches, proteins scores (as MudPIT score) were derived from ions scores as a non-probabilistic basis for ranking protein hits, disregarding all individual peptides with scores below 20. Only proteins that met the following criteria were accepted as unambiguously identified: 1) MASCOT score >76 (probability-based MOWSE score: -10*log(P), where P is the probability that the observed match is a random event; score > 76 indicate identity or extensive homology (P < 0 · 05)).

Because all protein hits derived from the MASCOT search did not satisfy the above reported criteria, all the MS/MS spectra were also subjected to manual interpretation assisted by the PepNovo software (http://proteomics.ucsd.edu/Software/PepNovo.html) in order to obtain peptide sequence information.

### Synthetic peptides

Two of the nine identified peptides, peptides H1 and H2, were purchased from CASLO, Lyngby, Denmark. The peptides were synthesized using Fmoc solid phase technology and the peptide content and purity were determined by high performance liquid chromatography (HPLC) and mass spectrometry (MS) analysis.

### Antimicrobial assays

#### ***Microbial strains***

The strains *Staphylococcus aureus* ATCC 25923, *Staphylococcus aureus* ATCC 29213, *Staphylococcus aureus* ATCC 6538, *Enterococcus faecalis* ATCC 29212, *Pseudomonas aeruginosa* ATCC 15442 were used in the assay. The good biofilm producer *Staphylococcus epidermidis* ATCC 35984 was also included in the analysis.

#### ***Minimum inhibitory concentrations (MIC)***

MICs of 5-HCC and of chemically synthesized peptides were determined by a micro-method as previously described (Schillaci et al. 2008; Schillaci et al. 2005). Tryptic Soy Broth (TSB, Sigma) containing 2% glucose was used as medium.

#### ***Biofilm capability evaluation (Safranin method)***

The staphylococcal strains and *P. aeruginosa* ATCC15442 were tested for their ability to form biofilms. Briefly, bacteria were grown in TSB containing 2% glucose overnight at 37°C in a shaking bath and then diluted 1:200 to a suspension with optical density (OD) of about 0.040 at 570 nm (Pitts et al. 2003). Polystyrene 24-well tissue culture plates were filled with 1 ml of diluted suspension and incubated for 24 hours at 37°C. The wells were then washed three times with 1 ml of sterile phosphate-buffered saline (PBS) and stained with 1 ml of safranin 0.1% v/v for 1 min. The excess stain was removed by placing the plates under running tap water. Plates were dried overnight in an inverted position at 37°C. Safranin-stained adherent bacteria in each well were re-dissolved to homogeneity in 1 ml of 30% v/v glacial acetic acid, and the OD was read at 492 nm. Each assay was performed in triplicate and repeated at least twice.

#### ***Interference with biofilm formation assay***

The procedure described above was used to evaluate the activity of synthesized H1 and H2 in the prevention of biofilm formation. Polystyrene 24-well tissue culture plates were filled with 1 ml of diluted bacterial suspension, obtained and diluted as previously described, and sub-MIC concentrations (6.2 and 3.1 mg/ml) of peptides 7 and 8 were directly added to the bacterial suspension at time zero and incubated at 37°C for 24 hours. The wells were then washed and stained with safranin as per the biofilm forming assay. By comparing the average optical density (O.D.) of the growth control wells with the sample wells, the following formula was used to calculate the percentages of inhibition for each concentration of the sample:

(1) $$\text{inhibition}\left(\%\right)=\frac{\mathrm{OD}\;\text{growth}\;\text{control}-\mathrm{OD}\;\text{sample}}{\mathrm{OD}\;\text{growth}\;\text{control}}\times 100$$

#### ***Hemolytic assay***

The assay was performed as described by (Arizza et al. 2013). In brief, freshly collected rabbit erythrocytes (RE) with heparin, kindly provided by the “Zooprophylaxis Institute of Sicily” (Palermo, Italy), were washed (400 g for 10 min at 4°C) to remove the buffy coat, and the erythrocytes obtained were washed three times with phosphate-buffered saline (PBS: 6 mM KH_2_PO_4_; 30 mM Na_2_HPO_4_; 0.11 M NaCl; pH 7.4) and suspended in 10 ml PBS to obtain an 80 × 10^6^ cells/ml suspension. Aliquots of 200 μl of RE suspension were mixed with 200 μl of H1 and H2 concentrations (1.5, 3.2, 6.2 and 50 mg/ml) prepared in PBS, two-fold sequentially diluted v/v with a PBS. After a 1 h incubation at 37°C, the reaction mixture was centrifuged at 800 g for 15 min at 4°C to remove debris and residual erythrocytes. The O.D. of hemoglobin release was measured spectrophotometrically at a 541 nm wavelength. Spontaneous hemoglobin release (0%) was estimated incubating the RE with PBS while the complete hemolysis (100%) was assessed incubating the erythrocytes in a solution of 0.1% Triton-X 100 in distilled water and the hemolysis percentage was calculated according to the following equation:

(2) $$\text{Hemolysis}\left(\%\right)=\frac{O.D.\mathrm{Hb}\;\text{release}\;\mathrm{in}\;\text{the}\;\text{reaction}\;\text{mixture}-O.D.\text{spontaneous}\;\mathrm{Hb}\;\text{release}}{O.D.\text{complete}\;\mathrm{Hb}\;\text{release}}\times 100$$

#### ***Statistical analysis***

Each experiment was performed in triplicate. The values were the mean of three assays ± SD. Significance was determined by using the Student's t-test and differences were considered significant at P < 0.05.

## Results

### Conformational analysis of 5kDa peptide fraction (5-HCC) components

With the aim of detecting the presence of novel antimicrobial peptides and determining their sequence, the 5-HCC fraction was subjected to capillary RP-HPLC-HPLC/nESI-MS/MS. The MS/MS data obtained were used to query the protein database of *Echinodermata* and to perform *de novo* peptide sequencing.

With this approach, nine principal peptides with molecular masses ranging from 805.5 to 2215.7 Da were identified. Their sequences are reported in Table 1.

**Table 1:** Chemical-physical characteristics and amino acid sequences of peptides detected in 5-HCC content

**Peptides**	**ESI-MS calculated MH + Da**	**Total net charge (pH 7)**	**Total hydrophobic ratio (%)**	**pI**	**Sequences, and hydrophobic amino acids on the same face (underlined)**
#1	805.5	-1.8	28.57	4.47	FTDESHA
#2	919.2	-0.6	50.00	6.48	LSELLHHA
#3	1003.4	-0.6	44.44	6.48	GVLSELLHH
#4	1017.4	-0.6	55.56	6.48	VLSELLHHA
#5	1074.4	-0.6	50.00	6.48	GVLSELLHHA
#6	1367.6	-0.4	41.67	6.77	HLGHHALDDLLK
#7	1389.5	+0.9	41.67	7.56	HLGHHALDHLLK
#8	1547.6	+0.9	42.86	7.56	ASHLGHHALDHLLK
#9	2111.3	-3.1	27.78	4.23	EVKPNLTEKIEDLSQQMD

By using an antimicrobial peptide database (Wang and Wang 2004), we observed that only two of the nine peptides, Holothuroidin 1 (H1) and Holothuroidin 2 (H2), identified as peptide #7 and #8, whose molecular weights are respectively 1389.5 and 1547.6 Da, were likely to be novel AMPs, due to their chemical-physical characteristics and similarity with already defined AMPs. They had a sequence of 12 and 14 amino acids (a.a.), respectively, and the H2 differed only for the first two a. a. (A^1^ and S^2^), which were not present in H1. They are cationic peptides mainly enriched by residues such as lysine hystidine and arginine, with a net charge of +0.9, calculated by using the protein calculator v3.3. (http://www.scripps.edu/~cdputnam/protcalc.html) and a deduced pI of 7.5 calculated by using a web tool (http://isoelectric.ovh.org/) (Table 1).

Both H1 and H2 peptides showed a similarity ≥ 35% respectively with protonectins, a peptide present in the venom of the neotropical social wasp *Agelaia pallipes pallipes*, with a potent antimicrobial action against both Gram-positive and Gram-negative bacteria (Mendes et al. 2004) (Table 2) and signiferins, a naturally occurring cationic peptide produced by an Australian frog, *Crinia signifera*, that showed a wide spectrum of activity against Gram-positive and -negative bacteria including *B. cereus, E. faecalis, L. lactis, L. innocua, M. luteus, S. aureus, S. epidermidis* and *S. uberis* (Maselli et al. 2004) (Table 3). The other identified peptides (#1-#6 and #9) in *H. tubulosa* are negatively charged and have very little likelihood of being AMPs.

**Table 2:** Sequence comparation of Holothuroidin 1 with other AMPs

**Peptides**	**Sequence**	**Similarity (%)**	**Species**
Holothurioidin 1	H L**G**HHA**L**DH**L LK **		*Holothuria tubulosa*
Protonectin	I L**G** + TI**L** + G**L LK**GL	42.85	*Agelaia pallipes pallipes* (Mendes et al. 2004)
Polybia-CP	I L**G** + TI**L** + G**L LK**SL	42.85	*Polybia paulista* (Souza et al. 2005)
Macropin 1	G F**G** + MA**L** + K**L LK**KVL	40.00	*Macropis fulvipes* (Slaninova et al. 2012)
Signiferin 2.2	IIGH LIKTA**L**GF**L**G**L**+	37.50	*Crinia signifera* (Maselli et al. 2004)
Temporin-1DRa	HFL**G**++T**L**VN**L**A**K**KIL	37.50	*Rana aurora draytonii* (Conlon et al. 2007)

**Table 3:** Sequence comparison of Holothuroidin 2 with other AMPs

**Peptides**	**Sequence**	**Similarity (%)**	**Species**
Holothurioidin 2	ASH**L**GHHA **L**DH**L** LK		*Holothuria tubulosa*
Frenatin 3	GLMSV**L**G + HAVGNV**L**GG**L** FKS	38.09	*Litoria infrafrenata* (Raftery et al. 1996)
Signiferin 2.2	IIGH**L**IKTA **L**GF**L**GL+	37.5	*Crinia signifera* (Maselli et al. 2004)
Signiferin 2.1	IIGH**L**IKTA **L**GM**L**GL+	37.5	*Crinia signifera* (Maselli et al. 2004)
Temporin 1Ja	ILP**L**VG+N L**L**ND+**L**L+	37.5	*Rana japonica* (Isaacson et al. 2002)

**Figure 1:**
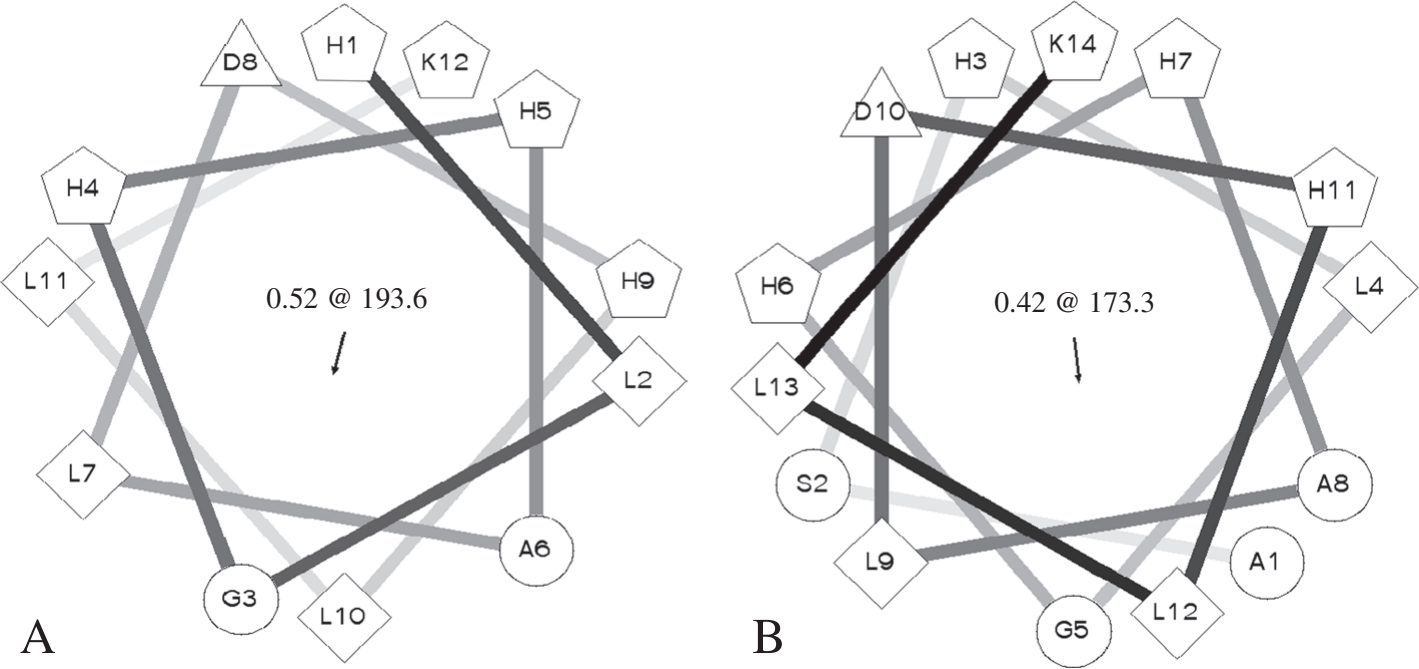
**Edmundson wheel projection of (A) Holothuroidin 1 and (B) Holothuroidin 2 calculated by the Helical Wheel Projection web tool.** Residues are schematically represented by geometric figures. Hydrophilic residues = circles, hydrophobic residues = diamonds, potentially negatively charged = triangles, and potentially positively charged = pentagons. In the centre mean relative hydrophobic moment and hydrophobic arc (@) are indicated.

A Schiffer-Edmundson helical wheel projection (Schiffer and Edmundson 1967) of H1 and H2, performed using Helical Wheel Projection (rzlab.ucr.edu/script/wheel/wheel.cgi), indicated that the α-helixes of both peptides had considerable amphipathic character, with the polar and mainly cationic residues D^8^, H^1^, H^4^, H^5^, H^9^ and K^12^ segregated on one polar face and the hydrophobic or non-polar residues A^6^, L^2^, L^7^, L^10^, L^11^ on the opposite apolar face for H1 (Figure 1A), and D^10^, H^3^, H^6^, H^7^, H^11^, and K^14^ segregated on the polar face and the hydrophobic or non-polar residues A^1^, A^8^, G^5^, L^4^, L^9^, L^12^, L^13^, S^2^ placed on the opposite apolar face for H2 Figure 1B). Their mean hydrophobic moments and hydrophobic arc size range were respectively 5.28 and 193.6° for H1 and 5.08 and 173.3° for H2 (Figure 1).

**Figure 2:**
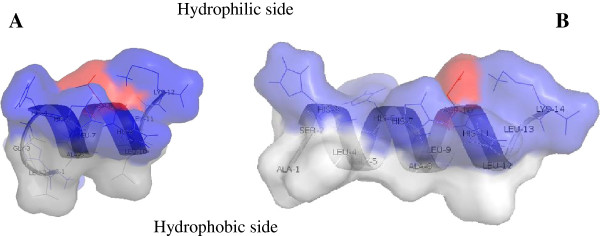
**A ribbon representation of Holoturoidin 1 (A) and Holoturoidin 2 (B).** The amphipathic nature of the peptide is shown in this representation with the hydrophilic side above and the hydrophobic side below the polypeptide backbone. The potential surface is superimposed. Color code: acidic residues in red, basic residues in blue, and hydrophobic residues in white.

The use of DeepView software (Swiss Institute of Bioinformatics - http://spdbv.vital-it.ch/) made it possible to resolve the secondary alpha-elical structure, which for both peptides was shown to be a periodic distribution of hydrophobic and hydrophilic residues, residues 4 – 10 for H1 and residues 2 – 12 for H2, in which the hydrophobic face is opposed to the hydrophilic one (Figure 2).

### Antimicrobial activity of 5-HCC

The antimicrobial activity of the 5kDa peptide fraction from coelomocyte cytosol (5-HCC) of *H. tubulosa* expressed as minimum inhibitory concentrations (MICs) against microbial reference strains are listed in Table 4. The 5-HCC resulted active against all tested reference microbial strains with MIC values ranging from 125 to 500 mg/ml.

**Table 4:** Antibacterial activity of 5HCC assayed by MIC

**Bacterial strains**	**MIC**
	**Values *in vitro *(mg**/**ml)**
*S. aureus* ATCC 29213	**500**
*S. aureus* ATCC 25923	**250**
*E. faecalis* ATCC 29212	**500**
*P. aeruginosa* ATCC 15442	**500**

### Antimicrobial activity of synthetic peptides

The synthetic peptides H1 and H2 were screened for their antimicrobial activity against the same group used for the evaluation of biological activity of 5-HCC. The H2 resulted active at 12.5 mg/ml against planktonic Gram-positive (staphylococcal reference strains and *E. faecalis* ATCC 29212) and against *P. aeruginosa* ATCC 15442. A similar activity at the same concentration was observed for H1 but it resulted not active at the maximum tested concentration against *S. aureus* ATCC 29213, and *E. faecalis* ATCC 29212. See Table 5.

**Table 5:** Antibacterial activity of synthetic Holothuroidin 1 and Holothuroidin 2 assayed by MIC

**Bacterial strains**	**MIC (mg/ml)**	
	**Holothuroidin 1**	**Holothuroidin 2**
*S. aureus* ATCC 29213	>12.5	12.5
*S. aureus* ATCC 25923	12.5	12.5
*S. aureus* ATCC 6538	12.5	12.5
*S. epidermidis* ATCC 35984	12.5	12.5
*E. faecalis* ATCC 29212	>12.5	12.5
*P. aeruginosa* ATCC 15442	12.5	12.5

### Interference with biofilm formation

The impact of H1 and H2 on the biofilm formation of two staphylococcal reference strains, *S. aureus* ATCC 25923, and *S. epidermidis* ATCC 35984, were evaluated at concentrations of 3.2 and 1.5 mg/ml (well below the MIC values obtained against the planktonic forms) by staining with safranin adherent bacteria. The H1 inhibited biofilm formation was 51.8% for *S. aureus* ATCC 25923 and 68.5% for *S. epidermidis* ATCC 35984 at 3.2 mg/ml; the inhibition was 37.9% for *S. aureus* and 58.2% for *S. epidermidis* at the lower concentration of 1.5 mg/ml. The same concentrations were used to analyze the effect of H2 on biofilm formation. Higher inhibitions of 57.7% and 73.8% at 3.2 mg/ml and 40.5% and 59.7% at 1.5 mg/ml were observed on *S. aureus* and *S. epidermidis* biofilms respectively.

**Figure 3:**
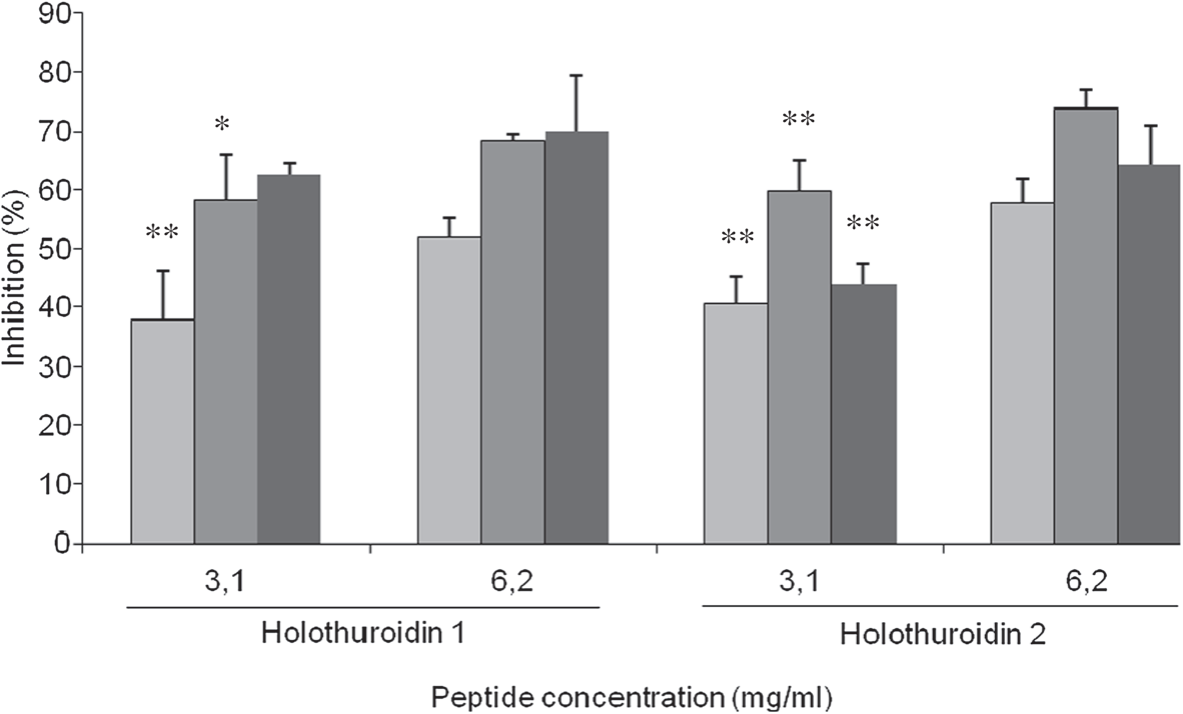
**Inhibition activity of Holothuroidin 1 and 2 on *S. aureus *ATCC 25923, *S. epidermidis *ATCC 35984 (light gray square symbol) and *P. aeruginosa *ATCC 15442 (dark gray square symbol) biofilms.** The data are expressed as the mean of three different experiments ± standard deviation. * = *p* < 0.05; ** = *p* < 0.01.

H1 and H2 also interfere with the biofilm formation of *P. aeruginosa* ATCC 15442 at the sub-MIC concentrations of 6.2 and 3.1 mg/ml. Inhibition percentages of 69.9% and 62.7% for H1 and 64.3% and 43.8% for H2 were observed (Figure 3).

### Hemolytic assay

**Figure 4:**
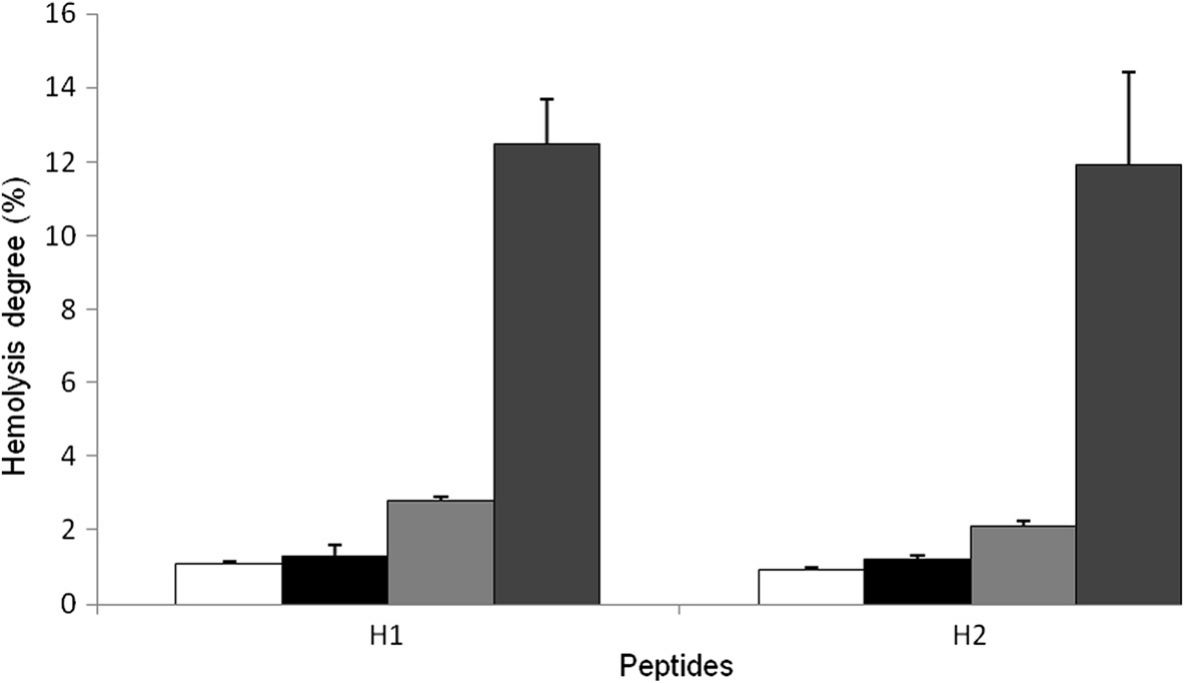
**Hemolytic activity of H1 and H2 peptides from *Holothuria tubulosa *hemocytes against rabbit blood cells at different concentrations: white square symbol = 1.5 mg/ml; Black square symbol = 3.2; gray square symbol = 6.2; dark gray square symbol = 50 mg/ml.** Data are the mean value of three separate experiments and expressed as percentage of hemolysis ± SD.

The hemolytic activity of antimicrobial peptides against mammalian erythrocytes is often used as to measure their cytotoxicity. The hemolytic experiment performed to evaluate the toxicity of the peptides H1 and H2 did not show a measurable toxic effect against RBC (~ 1%) at MIC concentrations (Figure 4). A slight hemolytic activity, about 12.5%, was evident only at the highest concentration (50 mg/ml) (Figure 4).

## Discussion

Antimicrobial peptides (AMPs) represent the first line of defense against pathogens in several organisms (Hancock and Lehrer 1998). The majority of AMPs, constituted by a short stretch of amino acids residues (10–100 maximum), present the following common features: broad antimicrobial spectrum, small sizes, a net positive charge due to an excess number of cationic residues, an over-representation of one or two amino acids (Hughes and Fenical 2010).

Recently, a variety of peptides with antimicrobial properties have been isolated from echinoderms (Andersson et al. 1983; Bryan et al. 1994; Ridzwan et al. 1995). Antimicrobial activity has also been reported in gonads of the asteroid *Marthasterias glacialis* (Stabili and Pagliara 1994). We have already demonstrated that echinoderms such as *Paracentrotus lividus* contain, in the low molecular weight fraction (<5kDa) of acid precipitate from their coelomocytes, peptides with antimicrobial activity against staphylococci antibiofilm (Schillaci et al. 2010; Schillaci et al. 2012).

In this study, the fraction of low molecular weight peptide (<5kDa) from coelomocytes of the *H. tubulosa* (5-HCC) showed broad antimicrobial activity against many Gram-positive and Gram-negative pathogens tested. The use of capillary RP-HPLC/nESI-MS/MS allowed the characterization in the amino acid sequence of nine principal peptides in the 5-HCC content. Only two peptides, #7 and #8, called respectively Holothuroidin 1 and Holothuroidin 2, of the total nine peptides found in 5-HCC, are likely to be AMPs (Wang and Wang 2004).

Structurally they share the characteristics of many antimicrobial peptides: they are cationic peptides and possess a significant proportion (~ 30%) of hydrophobic residues (Hancock and Lehrer 1998; Zasloff 2002). They may form, as secondary structures, an α-helical, which is the most common structure of AMPs in nature and which is essential, and often sufficient, for antimicrobial activity (Mor and Nicolas 1994; Skerlavaj et al. 1996; Storici et al. 1994; Tossi et al. 1994). The helixes of two peptides present an asymmetrical arrangement of the hydrophobic residues, specifically: four hydrophobic residues on the same face and a total hydrophobic ratio of 41.67% for H1, and for H2, a hydrophobic face opposite to the hydrophilic one and a total hydrophobic ratio of 42.86%. This characteristic conformation of the peptides, in the course of microbial membrane invasion, allows the non-polar face of their α-helical structure to interact with the membrane lipid core while at the same time permitting its hydrophilic face to engage in electrostatic interactions with the membrane lipid headgroup region (Phoenix et al. 2002).

Both peptides are not long enough (when in an alpha-helical conformation) to traverse the lipid bilayer of a bacterial cell. Indeed, the `barrel stave' mechanism requires a minimum of 20 residues (Bechinger 1997; Epand et al. 1995; Shai 1995), so probably some aggregation of Holothuroidin molecules on the membrane surface may be needed before the bacterial cell wall can be breached, e.g. via the so-called “carpet” mechanisms for the disruption of the bacterial membrane (Epand et al. 1995; Shai 1995).

In our study we observed that synthetic H1 and H2 did not show, in PBS, hemolytic activity, probably because the high ionic strength of PBS allows negatively charged sialic acid present on the erythrocyte membrane to neutralize the peptides. On the basis of this evidence, they could be classified as peptide antibiotics (Saberwal and Nagaraj 1995). The two peptides showed a modest broad-spectrum activity against planktonic strains (MIC equal to 12.5 mg/ml). However, they showed a good inhibition of biofilm formation *in vitro* at lower MIC concentrations when evaluated against the planktonic forms on two staphylococcal reference strains and *P. aeruginosa* ATCC 15442. Such additional antimicrobial effect could be due to different mechanisms of action during the biofilm formation: a) an interference with the initial adhesion of microbial cells to the surface could be due to modifications of the microbial membranes, b) the killing of the early bacterial colonizers, or c) the inhibition of Quorum Sensing (QS), i.e. the intercellular communication system involved in biofilm formation (Batoni et al. 2011).

Whatever the supposed mechanism of action, the prevention of biofilm formation – rather than its elimination – is an interesting strategy to contrast the growth, as a sessile community, of many pathogens.

The extensive clinical use of classical antibiotics has led to the growing emergence of many medically relevant resistant strains of bacteria biofilm (Patel 2005). Therefore, the development of a new class of antibiotics has become critical. The cationic antimicrobial peptides could represent such a new class of antibiotics (Andreu and Rivas 1998; Hancock 1997; Sitaram and Nagaraj 2002). The development of resistance to membrane active peptides whose sole target is the cytoplasmic membrane is not expected because this would require substantial changes in the lipid composition of cell membranes of microorganisms (Hancock 2001).

Novel AMPs derived from coelomocytes of *H. tubulosa* are attractive candidates for developing useful antimicrobial agents against surface adherent staphylococci and *P. aeruginosa* of clinical interest.

The anti-adhesion properties of the peptides H1 and H2 could be useful to contrast staphylococcal and *P.aeruginosa* biofilms involved in food spoilage and biofilm formation of food transmitted pathogens. Moreover, the tested peptides are a good starting point to design new synthetic derivatives with modified chemical-physical properties, with the aim to improve their antimicrobial activity against pathogens and their pharmaceutical potential (Brogden and Brogden 2011; Huang et al. 2010).

## Competing interests

The authors declare that they have no competing interests.
